# Improved Wavelength Calibration by Modeling the Spectrometer

**DOI:** 10.1177/00037028221111796

**Published:** 2022-07-13

**Authors:** Dongyue Liu, Bryan M. Hennelly

**Affiliations:** ^1^Department of Electronic Engineering, 8798 Maynooth University, Kildare, Ireland; ^2^Department of Computer Science, 8798 Maynooth University, Kildare, Ireland

**Keywords:** Wavelength calibration, Czerny–Turner spectrograph, transmission spectrometer, reference lamp

## Abstract

Wavelength calibration is a necessary first step for a range of applications in spectroscopy. The relationship between wavelength and pixel position on the array detector is approximately governed by a low-order polynomial and traditional wavelength calibration involves first-, second-, and third-order polynomial fitting to the pixel positions of spectral lines from a well known reference lamp such as neon. However, these methods lose accuracy for bands outside of the outermost spectral line in the reference spectrum. We propose a fast and robust wavelength calibration routine based on modeling the optical system that is the spectrometer. For spectral bands within the range of spectral lines of the lamp, we report similar accuracy to second- and third-order fitting. For bands that lie outside of the range of spectral lines, we report an accuracy 12–121 times greater than that of third-order fitting and 2.5–6 times more accurate than second-order fitting. The algorithm is developed for both reflection and transmission spectrometers and tested for both cases. Compared with similar algorithms in the literature that use the physical model of the spectrometer, we search over more physical parameters in shorter time, and obtain superior accuracy. A secondary contribution in this paper is the introduction of new evaluation methods for wavelength accuracy that are superior to traditional evaluation.

## Graphical abstract

**Graphical Abstract:**
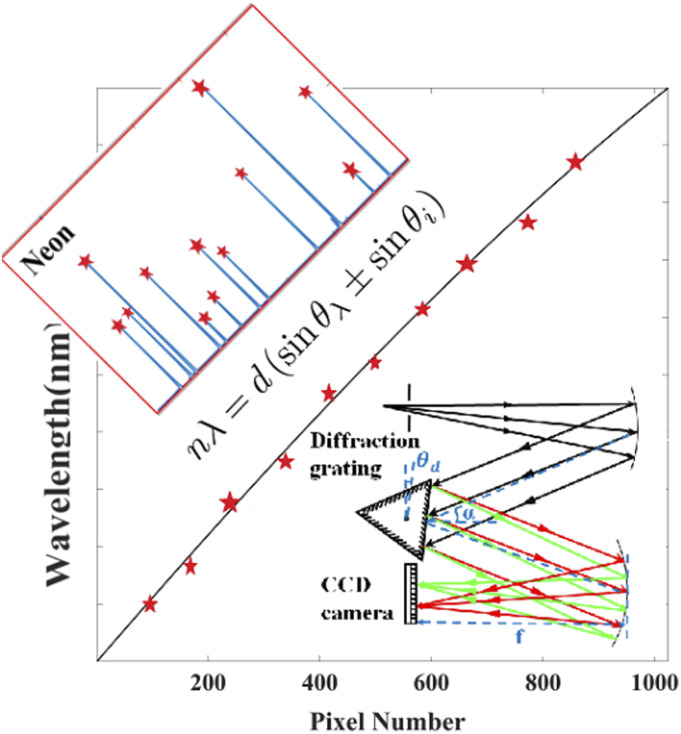

## Introduction

Wavelength calibration is an important first step various applications including astronomy,^1^ multi-spectral imaging,^2,3^ and optical coherence tomography (OCT).^4,5^ Another application, near-infrared spectroscopy (NIRS), which has widespread application in the identification of chemicals and biological materials,^6–8^ requires wavelength calibration in order to produce reliable classification of spectra.^9,10,11^

Of particular importance in the context of wavelength calibration is Raman spectroscopy. Like NIRS, Raman spectra are commonly used to identify and classify materials based on large datasets of known spectra. Applications include pharmaceutical manufacture and bioprocess monitoring,^12,13^ material science,^14^ and applications in clinical biology.^15,16^ Raman spectra have significantly higher resolution than NIRS spectra and, therefore, spectra must be subject to careful wavenumber and intensity calibration before comparison with a database. Wavelength calibration is commonly a first step in both wavenumber and intensity calibration.^17–19^

Typically wavelength calibration involves polynomial fitting of the two dimensional dataset that is the known reference lamp spectral lines(wavelengths) and the position that these are found on the detector (pixels).^17,18,20–22^ However, these methods tend to suffer from high error for regions outside of the spectral lines in the reference lamp, and this problem may be exacerbated for spectral bands for which there are few spectral lines available. Recently, there has been interest in using a physical model of the optical path in the spectrometer for the purpose of wavelength calibration,^23–27^ which overcomes this limitation. All of these methods use the grating equation as the basis for developing an equation that relates the wavelengths and pixels in terms of the system parameters such as the grating period, spectrograph deviation angle, grating angle, camera pixel size, and tilt. Some methods develop a system of simultaneous equations based on a set of wavelength, pixel pairs, and some are based purely on a brute-force search over the various parameters in order to find the best fit of the equation to an available set of wavelength, pixel pairs.

In this paper, we propose an algorithm based on the physical model that includes a brute-force search for some of the system parameters, while performing polynomial fitting within that search to account for others. In doing so, we significantly reduce the scope of the search and improve the overall accuracy of the method. The reported accuracy is better than previous papers in this area. In addition, we provide several new evaluation methods that go much further than any previous publication in the area of wavelength calibration and we rigorously compare performance against polynomial fitting methods over large datasets. A more detailed list of the specific contributions in this paper is provided in the next section following a review of the background.

## Background

### Wavelength Calibration Using Polynomial Fitting

Wavelength calibration of a spectrometer using a detector array is based on exploiting the relationship between wavelength and pixel position across the detector using wavelength reference standards, such as neon or krypton, which have well defined peak wavelengths.^28,29^ Typically, this involves fitting a low-order polynomial to pixel position and wavelength coordinates for a series of known peaks in the reference. The use of linear and higher order polynomials has previously been applied for this purpose; the selection of polynomial order varies in the literature on a case by case basis. Here, we provide a brief review of the key contributions in this area in recent decades, and in later sections the contribution proposed in this paper is described in the context of this background material.

In the late 1980s, Hamaguchi proposed a method for the calibration of Raman spectrometers.^17,18,30^ At that time, the use of “multi-channel detectors” was relatively new and included instruments such as silicon-intensified target tubes, intensified photo-diode arrays (IPDA), and early-stage charge- coupled devices (CCD) with limited extent. The basis of Hamaguchi’s approach was to first perform wavelength calibration using a wavelength standard such as neon, followed by conversion to wavenumber, making use of the laser wavelength in this calculation. In the simplest case, in the absence of distortion, a linear relationship between “pixel” and wavelength was assumed and a least-squares approach was proposed in order to achieve accurate calibration using only a few neon peaks. However, it was also emphasized in this work that “optical distortion” caused by spherical aberration in the spectrometer, or by the detector itself, such as in the case of an electrostatic-type IPDA, could result in a pincushion effect and a non-linear relationship between wavelength and position in the recorded spectrum.

Hamaguchi proposed a solution to this problem, whereby a wavelength standard with many peaks (such as a neon lamp in an appropriate band) could be recorded and a higher order polynomial could be used to describe the relationship between the recorded peak (distorted) positions, and the expected peak positions. Thereafter, recorded spectra would be first corrected for the non-linearity caused by the optical distortion by using this predetermined higher order polynomial to cast the spectrum into a “virtual channel”. Following this, a linear-relationship between wavelength and position could be assumed, facilitating a least-squares fitting of straight-line approach to calibration as in the simple case in which no distortion was present. Importantly, Hamaguchi notes that in the case of a non-linear relationship between wavelength and position, a large number of reference peaks are required and, furthermore, the reference spectrum should contain peaks close to each end of the spectrum, since a least-squares approach with higher order terms will often provide erroneous results when the fitted curves are extrapolated to regions where no data points are available.

In this paper, we also propose to account for the non-linearity of wavelength and pixel positions using a non-linear relationship; however, we do not limit ourselves to the use of fixed order polynomials. Instead, we model the spectrometer using basic diffraction theory and ray optics in order to derive the non-linear relationship. Like the Hamaguchi method, we cast the recorded wavelength-pixel positions of several neon peaks using this non-linear relationship, such that the relationship between wavelength and position becomes linear, followed by least-squares fitting of a straight-line. This method accounts for non-linear dispersion by the grating but does not attempt to account for optical-distortion as for the case of the method described above.

Linear/first-order fitting has also been applied to splice together adjacent spectral bands^21,22^ and has also been applied as the first step in intensity calibration using a calibrated white lamp or florescence standard, which is used to correct for variation in spectral intensity caused by wavelength variable transmission of the optical elements in the spectrometer or the wavelength dependent efficiency of the grating.^22^ This method assumes a linear relationship between position and wavelength, which is approximately true over narrow spectral bands and for low dispersion gratings. Calibration using linear regression is known to produce errors as a consequence of the non-linear relationship between wavelength and pixel position, which becomes more pronounced for high dispersion gratings.^31–34^ For some applications, such as for splicing, and for intensity calibration, or indeed for calibrating low dispersion systems, these errors are small enough to have low impact. However, for more accurate characterization of wavelength positions, up to fifth-order fitting has been preferred in some cases.^21,35^

Tseng et al. established possibly the most widely adopted protocol for wavelength calibration of modern spectrometers.^21^ Included in this protocol is the use of first-order fitting as a means to stitch together adjacent spectral windows, as well as second-order fitting in order to obtain higher accuracy. This protocol also included a method to improve results by first interpolating the peak regions in the spectrum in order to obtain sub-pixel accuracy of peak position. The authors reported a standard-deviation in the calibrated wavelength positions of the neon peaks <0.005 nm for a 1800 lines/mm grating and a spectrometer with 0.64 m focal length.

Despite the better accuracy provided by second-order fitting, some groups have continued to use first-order fitting of wavelength and pixel position. Hutsebaut et al. have established a widely adopted protocol for the calibration of a Raman spectrometer.^19^ For intensity calibration, they record a neon wavelength standard followed by first-order fitting of the peak wavelengths and pixel positions. This is used as a first step in order to wavelength-calibrate a white light reference spectrum, which is subsequently used for the intensity calibration of a Raman spectrum recorded using the same spectrometer. Since the intensity of this reference is relatively smooth with respect to wavelength, the accuracy afforded by linear-fitting is sufficient. As an indicative value for the goodness of fit, the root mean square error (RMSE) was calculated by the authors to be 0.03 nm for the calibrated neon wavelength values.

Carter et al. proposed three methods of Raman wavenumber calibration,^36^ one of which is based on wavelength calibration using a neon reference with first-order fitting of peak wavelengths and pixel position, followed by wavenumber conversion using the known wavelength of the excitation laser. The authors argue that their approach is simpler to the protocol in Gaigalas et al.^21^ and is, therefore, more suitable for frequent re-calibration. First-order fitting of wavelength and pixel is shown to be sufficient for the calibration of relatively narrow Raman bands (100 cm^−1^) and the authors state that higher-order fitting would be preferable for wider bands as outlined.^21^

Gaigalas et al. employed first-order fitting of wavelength and pixel position as a first step for the intensity calibration of a broad spectrum, whereby many spectra are spliced together following repeated rotation of the grating.^22^ The spectra of interest are produced by a white lamp and a fluorescence standard. Wavelength calibration using krypton was applied in advance. It was observed that the errors in the wavelength calibration follow a “quadratic trend”, although no further investigation is applied since this has little impact on the accuracy of the intensity calibration.

Martinsen et al. developed a protocol to calibrate a spectrometer with poor resolution^37^ by using a filter to sequentially isolate single peaks in the wavelength reference, followed by calibration based on the recorded peaks. Of particular interest in the context of our work, is the use of a “constrained cubic” polynomial for wavelength calibration, whereby the relationship between wavelength and pixel position is assumed to be predominantly linear with the residual term described by a weak third-order polynomial. In Bocklitz et al.,^38^ a fifth-order polynomial was used to relate wavelength and camera pixel for a neon–argon lamp as part of a wavelength/wavenumber/intensity calibration routine for Raman spectra. To the best of our knowledge, this is the only instance of a polynomial order >4 being used in a wavelength calibration routine.

Recently, there have been some efforts to improve the accuracy of wavelength calibration by first improving the quality of the reference spectrum in advance of calibration.^39,40^ This pre-processing includes denoising, stray-light removal,^41^ and deconvolution for the purpose of compensating for the spatial frequency response of the spectrometer,^42–44^ as well as improved estimation of peak positions based on Voight or Lorenzian fitting.^39,45,46^

### Wavelength Calibration by Modeling the Physical System

Recently there has been interest in using a physical model of the optical path in the spectrometer for the purpose of wavelength calibration.^23–27,47,48^ In the first such method^23^ a wavelength calibration routine was developed based on modeling the optical system for the case of a Czerny–Turner spectrograph using reflective concave mirrors. Similar to the method proposed in this paper, this method uses the diffraction equation to derive a relationship between the detector pixel position and the wavelength. A series expansion is applied to this equation, and only the first three terms are used. The resulting expression is a second-order polynomial, the coefficients of which are defined in terms of the system parameters, including the grating angle, the deviation angle, the grating period, the focal length, the camera pixel size, and the tilt of camera. Assuming a known constant grating period, all of these parameters can be determined by a simple second-order polynomial fit applied and examination of the resulting coefficients. Effectively, this second-order polynomial fitting provides the basis for all future calibrations; using the parameters from the original second order fitting a single peak is sufficient to calibrate following thermal expansion, which affects the values of the focal length or deviation angle. Although this algorithm uses a model of the system, it is essentially a second-order polynomial fitting method and is subject to the same errors that can result from polynomial fitting, in particular when the reference spectrum does not have lines that cover the full wavelength bandwidth of the spectrometer. It should be noted that the proposed algorithm also takes into account changes in the reference wavelength due to variation in the refractive index of the air taken from Birch and Downs.^47^

In Liu and Yu,^24^ a wavelength calibration approach is proposed that also uses a physical model of the spectrometer, which replaces the need for polynomial fitting described above with a brute-force search. The relationship between pixel position and wavelength is described as a function of the various system parameters including the grating angle. A brute-force search over these parameters is applied in order to find the best fit for the recorded peaks from a reference neon lamp or similar. The key advantage of this approach is the accuracy of the calibration outside the end-peaks in the reference since polynomial fitting does will not extrapolate well in these bands, and also the ability of the method to be used with reference spectra containing only a small number of peaks. Liu and Yu proposed the first instance of this approach in 2013,^24^ and we provide a brief review of this work here, since it is most similar to the wavelength calibration algorithm proposed in this paper. A physical model of a Czerny–Turner spectrometer is used to derive the relationship between the three-dimensional coordinates of the camera port and the points at which the various wavelengths will come to focus. This physical model employs several system parameters relating to the four key elements in the spectrometer: (i) the angle of the collimating mirror, (ii) the angle of the grating, (iii) the angle and center position of the imaging mirror, and (iv) angle and center position of the detector. All of the aforementioned angles were taken to be one-dimensional, while the center locations of the latter two elements were considered in two dimensions. Of these eight parameters, only four were included in the calibration algorithm as variables: the angle of the grating, and the angle and center position of the detector. The remaining four parameters were assumed to be fixed and their values were measured. The calibration algorithm is based on a brute-force search in a predefined range over these four parameters, in order to find the set of parameters that provides the best fit for the recorded position-wavelength values.

Zhang et al.^25^ were particularly interested in developing a model that could also account for a grating that was mounted on a sine-bar to achieve rotation. The model included several parameters relating to the mechanical function of the sine-bar. The authors identified six key parameters, which were functions of the sine-bar mechanical properties, as well as the grating period, the angle of deviation, and the center of the detector. The wavelength calibration algorithm is based on solving a set of simultaneous equations that are derived from the physical model, in order to estimate the six key parameters. The authors state that this places a lower limit of five reference peaks for the the algorithm to work. However, it should be noted that such an approach may be adversely affected by error in estimating a single peak position, whereas the iterative approach described earlier^24^ would likely be significantly more robust to errors in a single peak position value.

In Yuan and Qiu,^48^ the authors use the diffraction equation to derive a single equation to relate pixel position and wavelength for a lens based reflection spectrometer. They identify three coefficients in this equation, each composed of two or more system parameters including focal length, deviation angle, grating angle, camera center, and pixel size. Solving for these three unknowns requires only three spectral lines from a mercury lamp. The authors report superior results compared with first, second, and third order polynomial fitting as well as two trigonometric’ methods. They report a “standard error” (which is similar in definition to RMSE except that it includes the number of coefficients used in the model in its definition) of 0.05nm.

In Bell and Scotti,^26^^,27^ a number of wavelength calibration algorithms are developed based on the physical model. This model accounts for all of the system parameters that are investigated by other researchers,^24,25^ but also accounts for tilt of the detector both horizontally and vertically, as well as accounting for displacement of the input irradiance vertically along the slit. As for other papers, the diffraction equation is used as the basis for deriving a model for the physical system. The algorithm fits up to nine system parameters to this equation although the method of fitting is not discussed. This is reduced to eight when the grating angle is known precisely using an optical encoder. With this encoder, the wavelength accuracy is reported to be 0.005nm and 0.025nm without. It should be noted that the proposed algorithm also takes into account changes in the reference wavelength due to variation in the refractive index of the air according to Ciddor.^49^

We also note that modeling the physical system has also previously been considered for Echelle spectrometers.^50–52^

### Contribution in this Paper

In this paper, a wavelength calibration method is proposed that is similar in design to that in Liu and Yu,^24^ with several differences.

• Similar to the method in Liu and Yu,^24^ our algorithm also searches over the variable parameters:
  (1) The grating angle, which can often be electronically controlled.
  (2) The center position of the camera with respect to the optical axis.

 However, the physical model presented here also accounts for several more system parameters, including small errors in:

  (3) The diffraction grating period and/or dispersion caused by displacement of the input irradiance spot vertically along the spectrometer slit. Image curvature is common in off-axis spectrometers^53^ and leads to a deviation in the effective grating period.^26,27^
(4) The angle of the optical axis with respect to a flat grating position.
(5) The focal length of the spectrometer.
(6) The camera pixel size
(7) A rotation of the camera plane.

We note that the latter item relates to in plane rotation of the camera. Rotation of the detector plane with respect to the optical axis cannot easily be accounted for in the proposed algorithm, and care must be taken, experimentally, in order to ensure that slight defocusing of the spectrum irradiance does not occur on the detector.^52,54^

• Although seven parameters are listed above, the algorithm proposed in this paper does not employ a brute-force search over all of these, which would be intractable. Instead, a brute-force search is applied over a limited range of values for parameters (1), (3), and (4), only. The remaining parameters are all estimated using a simple ordinary least-squares fitting of a first-order polynomial within the three-dimensional brute-force search. This is facilitated because parameters (2), (5), (6), and (7) will participate only in a simple shifting and scaling of the spectrum recorded by the detector. Therefore, the brute-force search space in our algorithm has one dimension less than that defined in Liu and Yu,^24^ while effectively searching over several more dimensions.
• The physical model is extended to account for spectrometers using both:
 (1)  A reflection grating. In this case, the physical model is based on a Czerny–Turner architecture. The resulting algorithm is tested on an Andor spectrometer with a rotating grating.
 (2)  A transmission grating. In this case, the model is adapted for a Kaiser spectrometer with fixed volume holographic phase grating.
• The performance of the wavelength calibration algorithm is thoroughly investigated across a variety of gratings with different periods.
• The algorithm is rigorously evaluated using several different methods including “leave-one-out” and “leave-half-out”, which provide a more accurate assessment of the calibration when compared to traditional approaches, particularly in spectral regions between the peaks and outside of end-peaks in the reference spectrum. Similar cross-validation approaches are commonplace in the field of chemometrics^55,56^ but we believe this is the first time they have been applied in the context of wavelength calibration.
• Finally, and most significantly, we report that the proposed method is significantly more accurate than any calibration method that we have so far reviewed in the literature for similar spectrometers. We report a standard deviation of <0.002 0.002 nm, which appears to be approximately independent of grating period and resolution. High accuracy is maintained outside of the end-peaks of the reference spectrum.

## Relationship Between Wavelength and Pixel Position in a Spectrometer

### Physical Model for Generalized Spectrometer with Rotating Grating

In this section, an equation is derived that relates the wavelength of a point-source at the spectrometer slit, to the position of the image of this point on the array detector. This derivation will form the basis of the calibration algorithm that is later developed in the following sections. The derivation is general for both transmission and reflection gratings, and the calibration algorithm can, therefore, be applied to spectrometers that employ both types of gratings as demonstrated in the subsequent subsections.

The diffraction grating is the main component of the spectrometer. The grating equation describes the relationship between the grating structure, the incident angle, and the angle of the diffracted light:

(1) $$n\mathrm{\lambda =}d\left(\sin\theta_{\lambda }\pm \sin\theta_{i}\right)$$

where d is the grating period, $\theta_{i}$ represents the angle of the incident ray of wavelength λ with respect to the grating normal, $\theta_{\lambda }$ is the angle at which this ray is diffracted, and n is the diffraction order. The ± term in the grating equation is negative for a transmission grating and positive for a reflection grating. Curvature of the slit image in the detector plane is caused by the displacement of the irradiance spot vertically along the slit resulting in an oblique angle of the light incident on the grating, and can be accounted for by adapting the grating equation as follows:

(2) $$\begin{array}{l} n\mathrm{\lambda =}d\cos\gamma \left(\sin\theta_{\lambda }\pm \sin\theta_{i}\right)\\ n\mathrm{\lambda =}d\prime \left(\sin\theta_{\lambda }\pm \sin\theta_{i}\right) \end{array}$$

where γ is the vertical oblique angle subtended by the optical axis and the line connecting the center of the collimating lens (or mirror) and the vertical position of the spot on the slit^26,27,53,57^ and ${d\;}^{\prime }=d\cos\gamma$.

**Figure 1.**
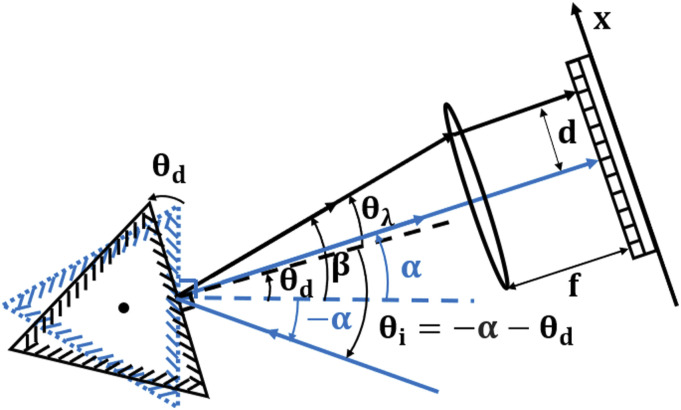
Diffraction of a ray by a rotated grating. The blue illustration shows the zero-order diffraction of an incident ray onto the optical axis of the spectrometer for a flat grating position. The black image shows the −1 order diffraction of the same ray following rotation of the grating by an angle $\theta_{d}$. We stipulate that the counterclockwise direction is positive for all angles.

Spectrometers often employ a rotating grating such that different wavelength bands can be projected onto a fixed detector. In the case that the grating is rotated by an angle $\theta_{d}$, both the incident and diffraction angles will be altered. This is illustrated in Fig. 1 in which a reflection grating is mounted on a rotating triangular base; this is similar to the design of one of the two spectrometers that is investigated later. The blue image represents the initial state of the spectrometer, without rotation, for which the incident ray is propagating at an angle α with respect to the grating normal. The zero-order diffracted ray (also at angle α with respect to the grating normal) propagates through the center of a lens of focal length f, and on to the center of a detector array; for simplicity, we will later refer to this as the optical axis of the spectrometer. We note that the value 2α is often referred to as the deviation angle of the spectrometer. We also note that the focusing optic can also takes the form of a parabolic mirror as described in the following section. The black image represents the state of the spectrometer following rotation of the grating by an angle of $\theta_{d}$. For the same incident ray we derive the position of the resulting $n^{th}$ order diffracted ray on the detector.

The grating equation can be rewritten to describe diffraction by the rotated grating as follows:

(3) $$\frac{n\lambda }{d}=\sin\left(\beta -\theta_{d}\right)+k\sin\left(-\alpha -\theta_{d}\right)$$

The parameter β in the figure represents the angle of the diffracted ray with respect to the grating normal for the initial state. The ± symbol has been replaced with the parameter k, which takes the value of +1 and −1 for transmission and reflection gratings, respectively. The angle β can be defined in terms of the other parameters as follows:

(4) $$\mathrm{\beta =}\sin^{\mathrm{-1}}\left[\frac{n\lambda }{d^{\prime }}-k\sin\left(-\alpha -\theta_{d}\right)\right]+\theta_{d}$$

The angle between the *n*th-order diffracted ray and the optical axis is β−α. The position at which this ray will be incident on the detector array is given by:

(5) xT=f⁡tan(β−α)+C

where C represents misalignment of the center of the detector array with respect to the optical axis and T is the pixel pitch in the detector. Equation 5 can be rewritten as follows:

(6) $$x=\frac{f}{T}\tan\left\{\theta_{d}+\sin^{-1}\left[\frac{n\lambda }{d\prime }-k\sin\left(-\alpha -\theta_{d}\right)\right]-\alpha \right\}+\frac{C}{T}$$

(7) $$\lambda =\frac{d\prime }{n}\left\{\sin\left[\tan^{-1}\left(\frac{xT-C}{f}\right)+\alpha -\theta_{d}\right]+k\sin\left(-\alpha -\theta_{d}\right)\right\}$$

Equations 6 and 7 form the basis of the calibration algorithms that are proposed in later sections. Before these algorithms are described, we first explore the nature of the relationship between the wavelength, λ, and pixel position, x, as defined by Eq. 6, for two spectrometers, which are later the subject of the proposed calibration algorithms. The first spectrometer of interest is a Czerny–Turner spectrometer employing parabolic mirrors and three different plane-ruled reflection gratings. The second is a lens based spectrometer employing a volume-phase holographic transmission grating. Both spectrometers are described in more detail below, followed by a discussion on the application of Eq. 6 to model each system.

### Reflection Spectrometer

**Figure 2.**
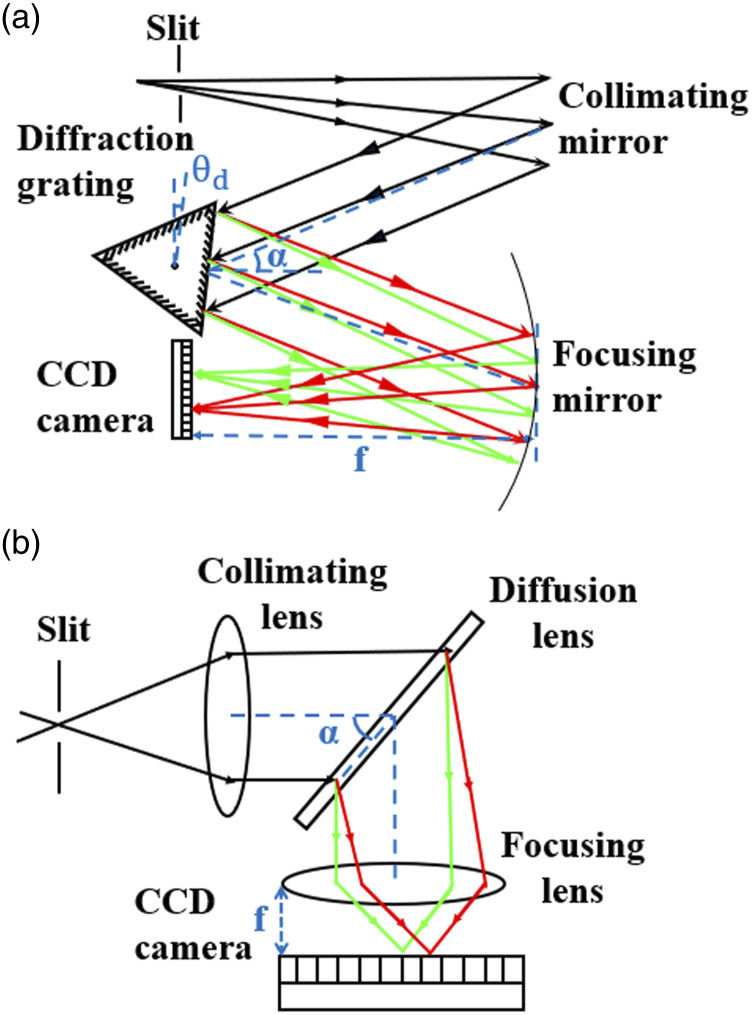
(a) The Czerny–Tuner spectrometer using parabolic mirrors and a rotating grating, and (b) a transmission spectrometer utilizing glass lens focusing and a holographic grating. The proposed wavelength calibration algorithm is general such that it can be applied to both types of spectrometers.

A traditional Czerny–Turner spectrometer with focal length 500 mm and with a motorized rotating grating was utilized for most of the experiments reported in this paper (Shamrock 500; SR-500i-A; Andor UK), which is illustrated in Fig. 2a.

Converging light enters the spectrometer slit and is collimated by a parabolic mirror and directed onto a grating, housed on triple grating turret and mounted on a rotation stage. The three gratings on the turret are all plane-ruled reflection gratings with the following specifications: 1000 lines/mm with blaze at 900 nm (Andor SR5-GRT-1000–0900; Andor UK), 600 lines/mm with blaze at 750 nm (Andor SR5-GRT-0600–0750; UK), and 300 lines/mm with blaze at 760 nm (Andor SR5-GRT-0300–0760; UK). The angled grating directs the n =−1 diffraction order towards a second parabolic mirror, which focuses the image of slit at the detector plane. The detector is a cooled CCD (Andor iDus; DU420A-BR-DD; UK) with 256 × 1024 pixels with a pixel-pitch *T* of 26 μm. Both parabolic mirrors have a focal length, f of 500mm and the half deviation angle, α, was measured to be 21.88°. The values of each parameter in Eq. 6 for this spectrometer are provided in Table I; grating angles, $\theta_{d}$ are selected for each grating.

**Table I.** The parameters for the two spectrometers illustrated in Fig. 2, which are investigated in this study. The parameters correspond to those in Eq. 6.

Parameter	Unit	Czerny–Turner	Trans-mission
Reflection/transmission *K*	–	+1	−1
Diffraction order *n*	–	−1	+1
Grating Period *d*	Lines/mm	300, 600, 1000	2455
Half the deviation angle α	Degrees	10.94	45
Grating angle θ_*d*_	Degree	4.8, 10.2, 17.2	0
Focal length *f*	mm	500	85
Camera pixel pitch *T*	μm	26	26
Camera width *N*	Pixels	1024	1024
Camera center position *C*	Pixels	0	0

### Transmission Spectrometer

A transmission spectrometer (Holospec-F/1.8I-VIS; Andor, UK) is also investigated in this study, the design of which is illustrated in Fig. 2b. Light is input to a slit of width 25 μm. A first lens collimates the light and is followed by a volume-phase holographic transmission grating with 2455 lines/mm (HS-HSG-532-LF; Andor, UK), which is angled at $\alpha \;\mathrm{=4}5^{\circ }$ with respect to the optical axis. A second lens captures the n=+1 diffraction order and images the slit onto the detector, a cooled CCD (Newton DU920P-BVF; Andor, UK) with 256 × 1024 pixels and pixel-pitch, T of 26 μm. Both lenses have a focal length of 85 mm. Notably, in this case, the diffraction grating is fixed and the grating angle is $\theta_{d}=0^{\circ }$.

## Relationship Between Wavelength λ and Pixel Position **x** for Both Spectrometers

In the sections that follow, a wavelength calibration algorithm is proposed that exploits the relationship between the wavelength, λ, and pixel position, x, for a given spectrometer, based on the model described above in Eq. 6. Here, we first explore the nature of this relationship for the reflection and transmission spectrometers that are described in the proceeding subsections.

**Figure 3.**
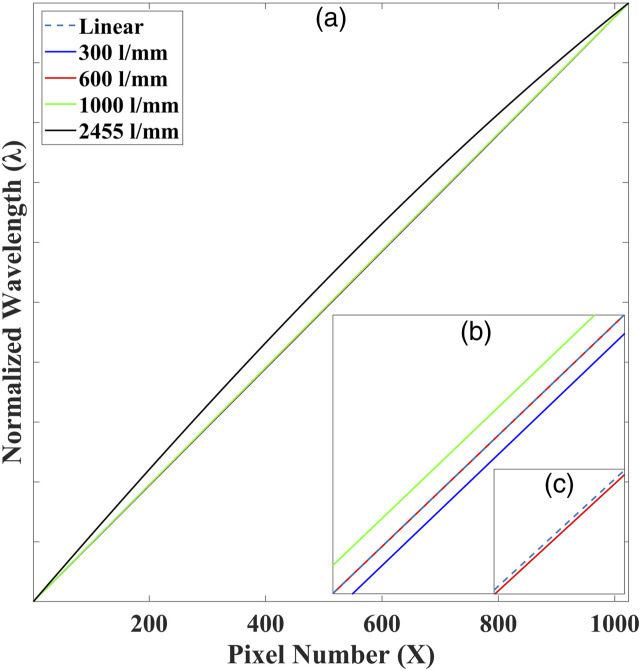
Investigation of the non-linearity of the (x,λ) relationship for the four different gratings that are later used for testing. (a), (b), and (c) show increasingly zoomed in areas. These plots are based on Eq. 6 using the parameters listed in Table II. The wavelength axis has been normalized for direct comparison.

This relationship is shown in Fig. 3, where the values in Table I are substituted into Eq. 6 for all four gratings. For ease of comparison, the wavelength axis has been normalized such that the minimum and maximum values appearing on the extreme ends of the CCD are 0 and 1 for all four diffraction gratings. A dashed line shows a linear relationship between x and λ. Interestingly, the 600 lines/mm grating is shown to exhibit the most linear relationship between x and λ, while the 300 and 1000 lines/mm gratings are both less linear but appear on opposite sides of the straight line. The 2455 lines/mm grating is significantly non-linear primarily owing to the shorter focal length and higher dispersion. The variability in the linearity of the relationship as a function of grating period and focal length may explain why several different polynomial orders have previously been proposed to be optimal for fitting as a means of wavelength calibration by different authors as discussed in the introductory section above.

## Calibration Based on the Physical Model

Here, we describe the sequence of steps that comprise the proposed wavelength calibration algorithm which is general to either the transmission or reflection spectrometer. We begin by clearly posing the problem and this is followed by describing two algorithms that can be used solve this problem.

*S* is the set of parameters that define the system as described earlier:

(8) $$S=\left[f,T,C,d^{\prime },\alpha \mathrm{,\theta}\right]$$

The relationship between the pixel coordinates on the detector, and the corresponding wavelength values that these pixels capture, is predicted by the physical model defined in Eq. 6 in terms of the parameter set *S*. This equation, and its inverse given by Eq. 7, which relates wavelength to pixel, are summarized by the following equations:

(9) $$\begin{array}{l} x=\mathrm{model}\left(S,\lambda \right)\\ \lambda =\mathrm{mode}l^{-1}\left(S,x\right) \end{array}$$

We move now from a continuous model to a discrete one, where the values of x and λ belong to the two sets of discrete values defined as follows:

(10) $$\begin{array}{l} X_{0}=\left[x_{1}^{0},x_{2}^{0}\mathrm{,\ldots ,}x_{N}^{0}\right]\\ \lambda_{0}=\left[\lambda_{1}^{0},\lambda_{2}^{0}\mathrm{,\ldots ,}\lambda_{N}^{0}\right] \end{array}$$

where $\lambda_{0}$ is a set of known neon peak wavelengths of which there are N, and $X_{0}$ is the corresponding set of positions in the detector plane at which these peaks are detected; we note that the values of $X_{0}$ will not in general be integers; a pre-processing step is first implemented in order to estimate the sub-pixel position of a peak as described in the following section.

The goal is, therefore, as follows. We wish to design an algorithm that can determine the set of parameters, S, that “best” relate the known N wavelength values, $\lambda_{0}$, and corresponding pixel positions at which these wavelengths were measured, $X_{0}$, according to Eq. 9. We begin by defining a brute-force algorithm based on Eq. 9 that is conceptually simple but computationally intractable. This algorithm is used as the basis of a second algorithm, which is significantly more computationally efficient.

Algorithm 1 Brute force. The first algorithm is based on a simple but computationally expensive brute-force search overall of the parameters in S and is made up of three steps:

(1) The first step provides initial estimates of the key parameters in S, which are defined as $S_{0}=\left[f_{0},T_{0},C_{0},d_{0},\alpha_{0},\theta_{0}\right]$ as follows:
  - The values of $\left(f_{0},T_{0},d_{0}\right)$ can be taken from the manufacturers specifications for the spectrograph and detector, where we assume $d_{0}=d$ and γ=0.
  - *α*_0_ is measured manually.
  - *C*_0_ and $\theta_{0}$ are estimated using a brute-force search over only these two variables for a single peak pair $\left(x_{i}^{0},\lambda_{i}^{0}\right)$.
(2) The second step is to perform a brute-force search over all six parameters in S over some range/step-size centered at $S_{0}$. Each unique set of parameters, $S_{j}$, in this range will produce set of pixel positions $X_{j}$ as follows:

(11) $$X_{j}=\mathrm{model}\left(S_{j},\lambda_{0}\right)$$

The specific set of parameters, $S_{\min}$, that produces the set $X_{\min}$ that most closely match the actual pixel values $X_{0}$ at which the peaks are detected are taken to be the true system parameters. This is determined by minimizing the error function defined in Eq. 12 over all parameter sets j in the range of the search.

(12) $$err=\sum_{i\mathrm{=1}}^{N}{\left(x_{i}^{j}-x_{i}^{0}\right)}^{2}$$

We acknowledge that a tight-grid brute-force search over such an error metric would not normally be applied since more efficient search algorithms are far more efficient such as steepest descents and simplex searching. Algorithm 1 serves only as a natural introduction to Algorithm 2, which must employ a brute-force search albeit over a much smaller range of values, and for this reason it is defined in terms of a brute-force search algorithm.

(3) Now that the system parameters $S_{\min}$ have been found, these can be used to relate the integer pixel (center) positions to the corresponding wavelength values, thereby providing wavelength calibration for the spectrograph. This third and final step is defined in the equation below:

(13) $$\lambda_{\mathrm{cal}}=\mathrm{mode}l^{-1}\left[S_{\min},\left[1\mathrm{,2,\ldots ,1024}\right]\right]$$

where $\lambda_{\mathrm{cal}}$ represents the set of calibrated wavelength values associated with each pixel center position. [1, 2, …, 1024] denotes the integer set of pixels.

While this algorithm provides for accurate calibration, it requires a brute-force search over six parameters and is computationally intractable. Even making the somewhat reasonable assumption that the specifications for $d^{\prime }=d$ and T, the camera pixel size, are without any error, will require a four-dimensional search, which remains time-consuming.

Algorithm 2 Speed-Up Using Least-Squares

In this section we attempt to speed-up the running time of Algorithm 1 by using the classical least-squares algorithm. Referring to Eq. 7, it is clear that the parameters f,T,C perform only scaling and additive functions on the spatial coordinate x and can, therefore, be accounted for using linear regression. Therefore, a brute-force search is required only over the remaining parameters α, d, and θ. The second algorithm also contains three steps as follows:

(1) This is identical to Step 1 in Algorithm 1.
(2) Here, a brute-force search is performed over only a three parameter set, $\left[\alpha ,d^{\prime },\theta \right]$, over some range of values, centered at $\left[\alpha_{0},d_{0},\theta_{0}\right]$ and using the values $f_{0},T_{0},C_{0}$ in order to provide an intermediate result.

(14) $$X_{j}=\mathrm{model}\left(\left[\alpha_{j},d_{j},\theta_{j},C_{0},f_{0},T_{0}\right],\lambda \right)$$

For each unique set of values $\left[\alpha_{j},d_{j},\theta_{j}\right]$ over the search range, the resultant values $X_{j}$ are linear-regressed with respect to positions at which the peaks were detected, $X_{0}$, in order to account for errors in f, T, and C, which provides an updated set of estimated positions $X_{j}$. For simplicity, we describe this operation in terms of the Matlab functions, polyfit and polyval, which are used to implement it as follows:

(15) $$\begin{array}{l} P_{j}=polyfit\left(X_{j},X_{0},n\right)\\ X_{j}=polyval\left(X_{j},P_{j}\right) \end{array}$$

where the function polyfit returns the coefficients of degree n that is the best fit (in a least-square sense) to describe the transformation between $X_{j}$ and $X_{0}$. This is followed by the function polyval, which applies this transformation to $X_{j}$ using these coefficients in order to provide the updated values for $X_{j}$.

     The specific set of parameters, $S_{\min}=\left[\alpha_{\min},d_{\min},\theta_{\min},f_{0},T_{0},C_{0}\right]$, and linear regression defined by $P_{\min}$ that produces the set $X_{\min}$ that most closely match the actual pixel values $X_{0}$ at which the peaks are identified and are taken to be system parameters. Once again, this is determined by minimizing the error function defined in Eq. 12 over all parameter sets j in the range of the search.

(3) Now that the system parameters $S_{\min}$ have been found, as well as the coefficents for the linear regression that accounts for error in f, C, and T, the integer pixel (center) positions can be related to the corresponding wavelength values, thereby providing wavelength calibration for the spectrograph. In this final step, the pixel position are projected into the wavelength domain by using the opposite process outline in Step 2:

(16) $$\lambda^{\prime }=\mathrm{mode}l^{-1}\left[S,polyval\left(\left[1,2,\ldots ,1024\right]\right),P_{\min}\right]$$

Here, the camera pixels are defined in terms of integers 1→1024.

## Overall Calibration Procedure

As discussed in the previous sections, the core principle of wavelength calibration of a spectrometer is to first record a reference spectrum containing some number of sharp, symmetrical, and well defined, known peak wavelengths. The second step is to identify the pixel positions of the various peaks in the recorded reference spectrum, which can then be used in the third step, which involves fitting with either a low-order polynomial or the pixel-wavelength relationship defined by a physical model. In either case, a matching wavelength value must be assigned to each pixel in the detector. The minimum number of requisite peaks in the recorded reference spectrum depends on the fitting method; a first-order polynomial fitting requires only two peaks, with this number increasing with respect to the polynomial order used. For the two physical model based methods reviewed earlier, there is also a minimum number of peaks required; for example, the brute-force method in Liu and Yu^24^ can work with a minimum of four peaks, while the method based on simultaneous equations^25^ requires a minimum of five peaks. A simple rule of thumb is that there must be at least as many peaks in the reference as there are variables in the physical model or coefficients in the polynomial. In general, however, more accurate results are obtained by increasing the number of peaks in the reference. As well as requiring a large number of peaks, the distribution of these peaks must also be considered. As noted by previous authors,^17^ wavelength calibration using a polynomial order greater than one, will result in poor calibration for bands that lie outside the end-peaks at either side of the reference spectrum. This is because there are no peaks in these extreme regions that can constrain the polynomial coefficients. However, first-order polynomial fitting (which is rarely accurate to begin with) and fitting based on the physical model are more robust to these out-of-band calibration errors.

**Figure 4.**
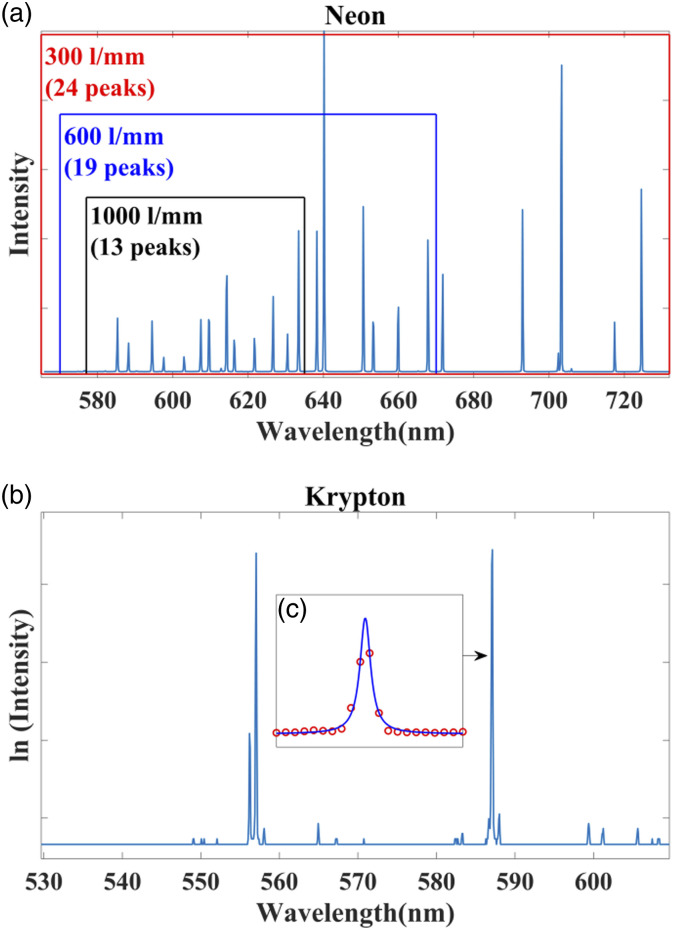
The spectrum of (a) neon (captured by the Czerrny–Turner spectrometer) and (b) krypton (captured by the transmission spectrometer). (c) A single krypton peak is shown illustrating the method of peak fitting for sub-pixel accuracy.

Typical reference lamps that are used for wavelength calibration include mercury–argon, neon, and krypton which are often selected based on the number of peaks available in the band of interest. The latter two reference lamps are utilized in this paper. In Fig. 4a, the spectrum of the neon lamp (Spectrum tube neon gas; Edmund Optics, UK) is shown, recorded using the Czerny–Turner spectrometer described earlier using a 300 lines/mm grating. Also shown in the figure are the bands of peaks that can be captured by the 600 lines/mm and 1000 lines/mm grating, which can be moved by rotation of the grating angle. In Fig. 4b the spectrum of the krypton lamp (Spectrum tube, krypton; Edmund Optics, UK) is shown, recorded using the transmission spectrometer described earlier using a 2615.8 lines/mm grating. In this case, the grating angle is fixed and the spectrometer can record only the band 530–610 nm. The bandwidth of this spectrometer and the Czerny–Turner spectrometer with the 1000 lines/mm grating are similar due to the significant different focal lengths in the two spectrometers. It is notable that wavelength calibration of this spectrometer with the krypton map with polynomial fitting with order two or more will result in significant error in the left-most band 530–556 nm due to the absences of peaks in this band. This “error band” would increase further using the neon lamp since the first useful peak occurs at 585 nm. In Table II the exact peak wavelengths for these two sources are shown, which have been taken from the database of the National Institute of Standards and Technology (NIST).^58^

**Table II.** Reference spectral lines used in this paper(with uncertainties^58^).

Wavelength/nm		
Neon		
585.2488 ± 5*e*-5	588.1895 ± 5*e*-5	594.4834 ± 5*e*-5
597.5534 ± 5*e*-5	602.9997 ± 5*e*-5	607.4338 ± 5*e*-5
609.6163 ± 5*e*-5	614.3063 ± 5*e*-5	616.3594 ± 5*e*-5
621.7281 ± 5*e*-5	626.6495 ± 5*e*-5	650.6528 ± 5*e*-5
630.4789 ± 5*e*-5	633.4428 ± 5*e*-5	638.2991 ± 5*e*-5
640.2248 ± 5*e*-4	653.2882 ± 5*e*-5	659.8953 ± 5*e*-5
667.8277 ± 5*e*-5	671.7043 ± 5*e*-5	692.9467 ± 5*e*-5
703.2413 ± 5*e*-5	717.3938 ± 4*e*-5	724.5167 ± 5*e*-5
Krypton		
556.2225 ± 5*e*-5	557.0289 ± 4*e*-5	558.0387 ± 5*e*-5
564.9562 ± 5*e*-5	567.2451 ± 5*e*-5	570.7513 ± 5*e*-5
583.2857 ± 5*e*-5	587.0916 ± 5*e*-5	587.99 ± 5*e*-5
599.385 ± 5*e*-5	601.2156 ± 5*e*-5	605.6126 ± 5*e*-5

In order to achieve accurate calibration, identification of the peak position requires sub-pixel resolution even though such a resolution is in general less than the specified resolution of the spectrometer. Various methods have been proposed in the literature to achieve such accuracy, including upsampling of the reference spectrum by zero-padding the discrete Fourier transform of the spectrum^19,21^ as well as fitting a Lorentzian function, or similar, to the pixel values in the region of the peak.^39,45,46^ We have tested these various approaches and determined that fitting with a Lorentzian function^59^ of the following form is slightly more accurate than upsampling:

(17) $$Peak^{\prime }=\frac{P_{1}}{{\left(x_{range}-P_{2}\right)}^{2}+P_{3}}+P_{4}$$

where Peak means $P_{1}$, $P_{2}$, $P_{3}$, and $P_{4}$ are fit-parameters, $x_{range}$ represents the pixel range of the peak, and $Peak^{\prime }$ represents the new intensity value in this pixel range.

An example of this approach is shown in Fig. 4c in which we show a Lorentz function that has been fit to one peak in the krypton spectrum. In the results section below, all of the peak positions in each reference spectrum are estimated with sub-pixel accuracy using this approach.

The overall procedure can be divided into four steps. The first step is to record the reference spectrum and the second step is to identify the sub-pixel position of each spectral peak that is listed in the related reference database as described above. The third step is the application of Algorithm 2 described earlier, which returns the parameters for a single equation that relates wavelength to pixel position. The final step is to apply this equation in order to identify the wavelength associated with the center of each pixel. For traditional calibration, the third step would be replaced with polynomial fitting to find the coefficients of an n-order polynomial and the fourth step would be application of this polynomial to identify the wavelength for each pixel.

## Experimental

### Recording of Reference Spectra

In total, we examine the performance of the proposed algorithm across two spectrometer designs and four different gratings periods as described earlier, each with varying dispersion. For the case of the Czerny–Turner system, only the reference neon lamp is applied and for the case of the transmission lens spectrometer, only the krypton lamp is applied; typical spectra are shown in Figs. 4a and 4b for both cases and in (c) an example of fitting the Lorentzian peak is shown around to the samples of a single krypton peak; this achieves sub-pixel accuracy as described in the previous section. The Czerny–Turner spectrometer is investigated using a 300 lines/mm, 600 lines/mm, and 1000 lines/mm corresponding to different wavelength bands as illustrated in Fig. 4a, while the transmission spectrometer uses a grating with 2455 lines/mm. For each of the three gratings in the Czerny–Turner spectrometer, 100 different reference spectra are recorded with slight movements of the grating rotation angle. For these three cases, a rigorous evaluation of the performance of the calibration is possible by calculating the ensemble average of the error metrics defined below, across the set of 100 reference spectra.

**Figure 5.**
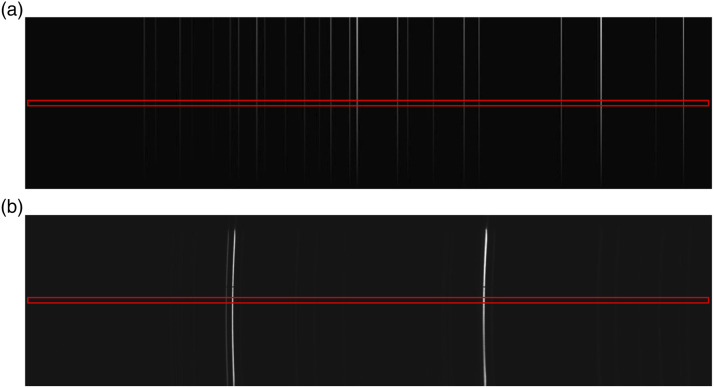
Image the reference spectra in the detector plane: (a) neon-300 lines/mm (b) krypton-2455 lines/mm; for the latter case clear distortion is observed due to the effect of the lens. A cropped row of pixels is extracted to mitigate this effect.

For all cases, the lamp was first carefully centered on the slit to ensure symmetrical spectral peaks. Andor Solis software is used to record the raw spectra in the image plane. Because of the strong irradiance from the lamps, a diffuser was positioned between the lamp and slit. To reduce the effect of noise, the accumulation time was varied to provide a photon count that was just less than the saturation level of the CCD. Rather than use Full Vertical Binning, which can produce error in the presence of image distortion, images were recorded from the detector as shown in Fig. 5 for both the (a) neon and (b) krypton lamps. The center row of pixels was cropped as illustrated by the red box in the figures. This approach was taken instead of Full Vertical Binning, in order to overcome the problem of image distortion as described earlier.

### Error Metrics

For comparison with similar methods proposed in the literature, several different error metrics are reported including, mean absolute error (MAE), the standard deviation (SD), and the root-mean-square-error (RMSE), all of which have appeared in different papers. These three metrics are defined below. A calibrated reference peak wavelength is denoted as $\left(\lambda_{i}\right)$, and assuming N such reference peaks exist in the reference spectrum, the following error metrics are defined:

(18) $$error\left(\lambda_{i}\right)=calibrated\left(\lambda_{i}\right)-NIST\left(\lambda_{i}\right)$$

(19) $$\mathrm{MΑE}=\frac{1}{N}\sum_{i=1}^{N}\left|error\left(\lambda_{i}\right)\right|$$

(20) $$\mathrm{RMSE}=\sqrt{\frac{1}{N}\sum_{i=1}^{N}{\left|error\left(\lambda_{i}\right)\right|}^{2}}$$

(21) $$\bar{\mathrm{SD}}=\sqrt{\frac{1}{N\mathrm{-1}}\sum_{i=1}^{N}{\left|error\left(\lambda_{i}\right)-\mathrm{ME}\right|}^{2}}$$

In order to provide a more reliable estimate of the error, the above metrics are calculated for a set of M different spectra where the grating is moved between captures. The ensemble average of each of the above metrics is calculated over these M reference spectra as follows:

(22) $$\bar{\mathrm{MAE}}=\frac{1}{M}\sum_{k=1}^{M}MAE\left(k\right)$$

(23) $$\bar{\mathrm{RMSE}}=\frac{1}{M}\sum_{k=1}^{M}RMSE\left(k\right)$$

(24) $$\bar{\mathrm{SD}}=\frac{1}{M}\sum_{k=1}^{M}SD\left(k\right)$$

### Evaluation Methods

We employ three methods of evaluation that employ the metrics listed above, two of which are proposed for the first time.

(1) *All peaks*. Here, all of the calibrated peaks from the reference are used in the error analysis. This is by far the most common approach in the literature.
(2) *Leave One Out Cross-Validation.* In order to remove any bias from the reference spectrum, we propose for the first time in the field of wavelength calibration (to the best of our knowledge) the use of cross-validation, an approach that is borrowed from the field of chemometrics.^55,56^ For the first case, “leave one out” cross-validation, one peak is removed from the reference spectrum used in the calibration process. The error metric is then applied only to this peak after calibration. This process is repeated for each peak in the spectrum and the average value for all cases is calculated. We believe that this is the first time that such an approach has been taken and we expect that it will provide a more accurate estimate of wavelength accuracy within the band of spectral lines provided by the reference lamp.
(3) *Leave Half Out.*. Similar to the approach taken in Liu and Yu^24^ we propose an evaluation based on calibrating using the left-most half of the reference peaks and applying the error metric to the right-most peaks of the calibrated spectrum. This is repeated using the right-most peaks for calibration and the left-most for error calculation. The average of the two values is taken. The advantage of this approach is that the accuracy of calibration is tested in bands outside of the outermost end-peaks in the reference lamp; the other two methods of evaluation only test for accuracy within the bounds of the reference spectrum lines.

### Comparison with Traditional Methods of Wavelength Calibration

In all cases, the proposed algorithm is compared with equivalent results from first-order, second-order, and third-order polynomial fitting and several interesting conclusion are made in the following section concerning the accuracy of these different methods under different conditions. Fourth-order fitting and higher provided no improvement in results and is not presented here.

## Results

**Figure 6.**
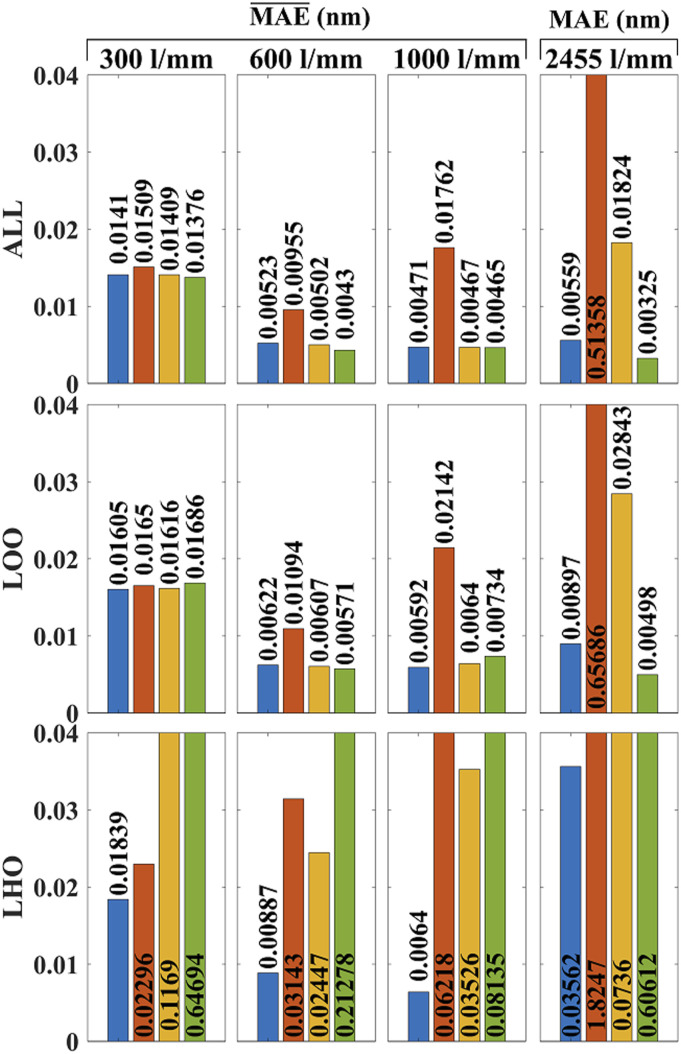
Evaluation of wavelength calibration accuracy using Mean Absolute Error. A neon reference lamp is used for the Crezny–Turner reflection spectrometer with three different gratings: 300, 600, and 1000 lines/mm and for these three cases the $\bar{MAE}$ error metric is applied over 100 spectra with grating movement between capture. A krypton reference lamp is used for the transmission spectrometer with grating 2455 lines/mm and for this case, the MAE error metric is applied over a single spectrum. The results of Algorithm 2, proposed in this paper, is given in blue and the results for first-, second-, and third-order polynomial fitting are given in orange, yellow, and green, respectively. The results of “all peaks” (ALL), LOO, and LHO are shown on different rows. For ease of comparison, the same axis range is used for all three evaluations. In several cases, the bars have been capped at 0.04 nm to improve visualization. The correct values are overlaid on the bars in all cases.

In this section, the results are presented for wavelength calibration using Algorithm 2 and compared with the corresponding set of results from first-, second-, and third-order polynomial fitting. These results are broken down into three sets of evaluations, corresponding to “all peaks” (ALL), “leave one out cross-validation” (LOO), and “leave half-out” (LHO). Furthermore, to facilitate comparison with other papers, which use various metrics, these evaluations are performed using three different metrics: $\bar{MAE}$, $\bar{RMSE}$, and $\bar{\mathrm{SD}}$ as defined in Eqs. 22–24. For the case of the transmission spectrometer, the grating angle could not be adjusted and so only a single spectrum was available. In this case the error metrics used in the evaluation are: MAE, RMSE, SD as defined in Eqs. 19–21. The results for the Mean Absolute Error metrics are shown below in Fig. 6.

It can be seen that the traditional evaluation method of inspecting all peaks provides approximately 10–20% superior results compared with LOO, which is proposed for the first time in this paper, and which we believe is a more accurate representation of wavelength calibration within the range of wavelength defined by the outermost reference lamp spectral lines. However, the overall trend of the results are the same for both ALL and LOO. It can be seen for both of these evaluation methods, that first-order fitting is the worst method in all cases but provides its best result for the 600 lines/mm grating, which was earlier shown to produce the most linear relationship between wavelength and pixel position (see Fig. 3). For the case of LOO evaluation, Algorithm 2 provides equivalent results to second- and third- order fitting for the 300, 600, and 1000 lines/mm gratings with very little difference between the three cases: (0.016 nm error for the 300 lines/mm case and 0.006 nm error for the other two). For the case of the 2544 lines/mm grating, which has by far the most non-linear relationship between wavelength and pixel position, third order fitting provides the best LOO accuracy with an error of 0.00498 nm, and Algorithm 2 provides the next best LOO accuracy with an error of 0.00897 nm. However, it should be noted that this case uses only a single spectrum and only 12 krypton peaks were available. More conclusive results could not be obtained by rotating the grating into different states as for the other three gratings.

The superiority of Algorithm 2 is evident for the third evaluation method, LHO, which provides a more accurate estimate of error in regions that are outside of the bandwidth of the reference lamp spectral lines. For the case of the 300 lines/mm reflection grating, Algorithm 2 provides the best LHO accuracy, with an error 0.01839 nm and first-order fitting is next best with an error of 0.02296 nm; second- and third-order fitting error are 6× and 35× worse than that of Algorithm 2, respectively. For the 600 lines/mm reflection grating, Algorithm 2 once again provides the best LHO accuracy with an error of 0.00887 nm; first-, second-, and third- order fitting errors are 2.5×, 3.5×, and 24× greater than that of Algorithm 2, respectively. For the third reflection grating of period 1000 lines/mm, Algorithm 2 once again returns by far the best LHO accuracy with an error of 0.0064 nm; first-, second-, and third-order fitting provide errors that are 10×, 4×, and 12× greater than that of Algorithm 2, respectively. Notably, when Algorithm 2 is used to calibrate the reflection spectrometer, LHO evaluation provides similar results when compared with LOO evaluation; there is only a marginal increase in error of 10–30% for the former, indicating that Algorithm 2 provides similar results far outside of the reference lamp spectral lines, as it does within the bandwidth of the lamp. This is not the case for the polynomial fitting; while third-order fitting provides equivalent results to Algorithm 2 for wavelengths within the bandwidth of the reference lamp (as evidenced by LOO evaluation), the error increases by a factor of 12–35 in regions outside of the lamp bandwidth (as evidenced by LHO evaluation).

For the case of the transmission grating with period 2455 lines/mm, all methods fare worse for LHO evaluation when compared with LOO evaluation; it can be seen that Algorithm 2, first-, second-, and third-order fitting provide LHO error that are 4×, 2.5×, and 121× greater than the corresponding LOO error. This is likely due to the small number of peaks available from the krypton lamp in the band of interest, which is exacerbated for LHO evaluation. Regardless, Algorithm 2 is the most accurate with an error of 0.03562 nm; first-, second-, and third-order fitting provide errors that are 51×, 2×, and 17× greater than that of Algorithm 2, respectively.

It is important to record the accuracy of the calibration methods in the context of the spectrometer resolution. The Czerny–Turner spectrometer with 300, 600, and 1000 lines/m grating is specified to have a resolution of 0.32 nm, 0.15 nm, and 0.09 nm, respectively, and the transmission spectrometer provides a resolution of 2.97 nm. All of these resolutions are significantly larger than the accuracy provided by Algorithm 2. Equivalent results are shown in the Appendix using standard deviation and RMSE in place of the MAE metric.

## Discussion

In terms of mean absolute error, the proposed algorithm is as accurate as polynomial fitting within the bandwidth of the reference lamp. Outside of this band third-order fitting has errors that are 12–35 times higher, while our algorithm has only 10–30% greater error.

It is difficult to directly compare the errors reported in previous papers on wavelength calibration accuracy. The main reason for this is that the various spectrometers that were used in other studies have highly varying wavelength resolutions due to different properties in terms of slit width, focal length, grating period, system distortion, and camera pixel size and noise characteristics. For this reason, we have chosen to compare the performance of the proposed algorithm directly with first-, second-, and third-order polynomial fitting rather than attempt to cross-compare with other studies. As an example, the (all-peaks) standard deviation error for second-order polynomial fitting over 10 neon spectra reported in one of the most cited papers^21^ is given as 0.005 nm. The spectrometer used in that paper was a Czerny–Turner spectrometer with a reflection grating with a higher resolution than the one used in this paper (focal length 0.64 m and grating 1800 lines/mm). The most similar result for our paper (second-order fitting, all peaks, 1000 lines/mm) has $\bar{\mathrm{SD}}\;\mathrm{=0}\mathrm{.00596}$ nm, taken over 100 neon spectra. For the two most similar methods in the literature that wavelength-calibrate using a physical model, much smaller accuracy is reported in Liu and Yu^24^ with a reported accuracy of 0.1 nm is reported. However, for this case the resolution of the 130 mm focal length spectrometer is signifcantly less than that of our own systems and is reported to be 0.5 nm at the central wavelength and up to 2 nm at the edge wavelengths. In Zhang et al.,^25^ the Czerny–Turner monochromator had a focal length of 300 mm, a grating density of 1200 lines/mm, and a 2160-pixel linear CCD detector with 14/μm pixel size. The authors report a mean absolute value error of 0.16 nm, and a standard deviation of error of 0.22 nm.

One should note that polynomial fitting algorithms are connected to the instrument modeling approaches because *sine* and *cosine* can be approximated as series expansions, and the terms of those expansions are closely approximated by cubic polynomials. This explains why the accuracy of third-order fitting and the proposed algorithm are similar for all cases within the region of the reference lines as evidenced by the leave-one-out evaluation.

## Conclusion

In this paper a novel wavelength calibration algorithm is proposed, which outperforms traditional polynomial fitting based methods, particularly in spectral bands that lie outside of the range of spectral lines provided by the reference lamp. Our method was demonstrated to be between 12–121 times more accurate that third-order fitting in such bands when compared to third-order fitting, and 2.5–6 times more accurate than second-order fitting. When compared to other recently proposed wavelength calibration algorithms that make use of a physical model of the system, the proposed algorithm is significantly faster and simultaneously fits to a larger range of physical parameters in the system, including distortion of the image plane. This is achieved by performing linear regression within the brute-force search for those parameters which linearly relate wavelength and pixel position on the detector.

A secondary, but nevertheless important, contribution in this paper is the introduction of a number of new evaluation methods for wavelength calibration accuracy. The traditional approach of evaluating error by inspecting each peak in the reference spectrum (ALL) is augmented with two approaches borrowed from chemometrics: leave one out cross-validation (LOO) and leave half out (LHO) evaluation. The former involves performing wavelength calibration using all but one of the reference peaks, and subsequently calculating error for that one peak. The same process is repeated for each peak. In this way the error of wavelength calibration for peaks within the spectral range of the lamp is better estimated since the peaks that are inspected were not part of the calibration process. LHO on the other hand provides a better estimate of accuracy outside of the range of spectral peaks in the reference lamp by using only one half side of the spectral lines for calibration, and the other half to calculate error. We believe that these metric should become the standard in evaluating wavelength calibration going forward.

In terms of future work, we believe there is scope to improve the proposed algorithm by obtaining a better first guess of the core spectrometer parameters in the search algorithm. This could be achieved by using the approach of Holy^23^ in which a set of simultaneous equations can be derived from the physical model to approximately solve for these parameters. Further, we believe better accuracy could be obtained if the spectral line positions for the reference lamp were corrected to account for the refractive index of air^47,49^ as has been done for other wavelength calibration methods; we made no attempt to do this in this paper. Finally, we believe that the proposed algorithm has potential as a first step for wavenumber calibration in Raman spectrometers. Accurate calibration is an essential first step in Raman based classification or chemical identification.^60,61^

## Figures and Tables

**Figure A1.**
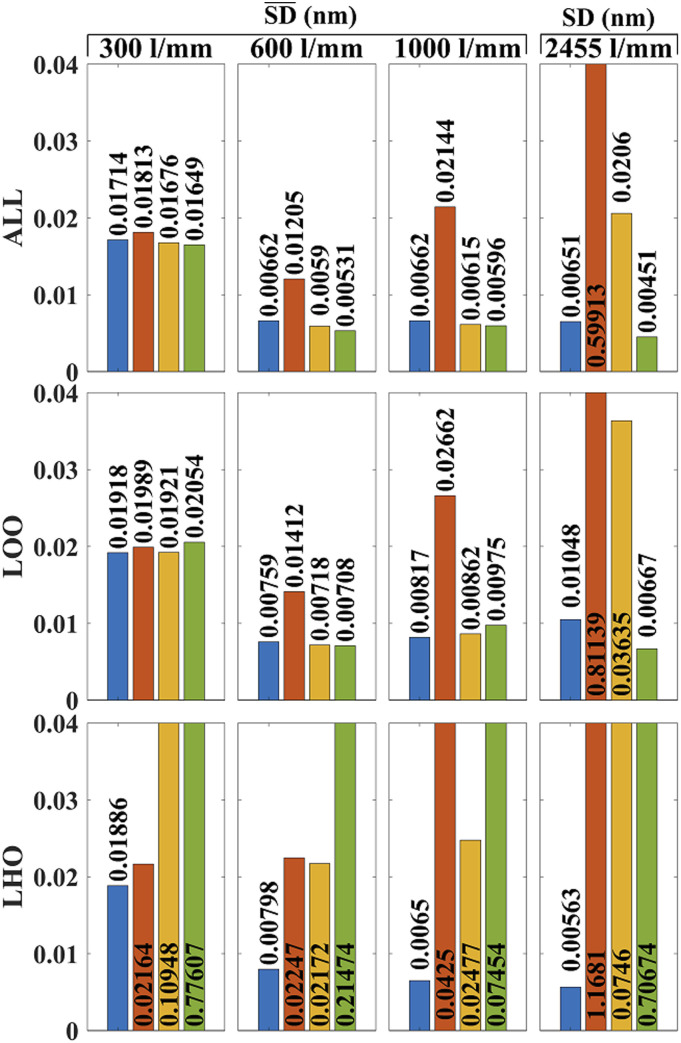
Standard deviation result for system.

**Figure A2.**
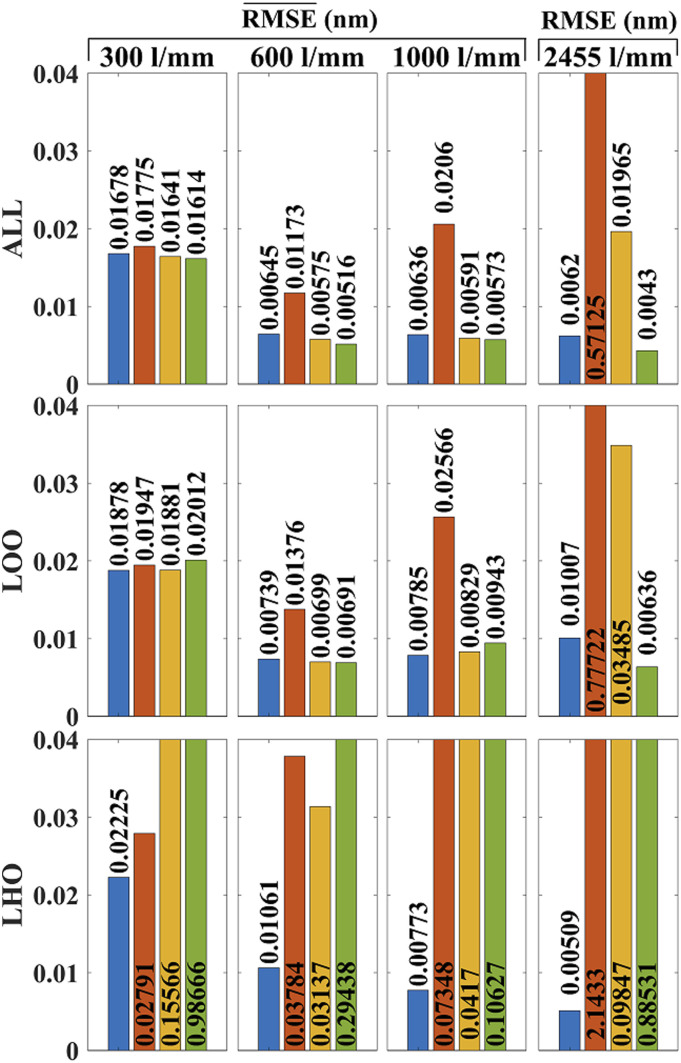
RMSE result for system.

## Appendix

In this appendix evaluation of the proposed algorithm is shown for the error metric of standard deviation (Fig. A1) and root mean sqaure error (Fig. A2) as defined above. These results correspond to those shown in Fig. 6 in the main body of the paper for the case of the error metric mean absolute error. These additional results are shown here to help in comparing with results from other papers.
